# Implementation of a structured paediatric admission record for district hospitals in Kenya – results of a pilot study

**DOI:** 10.1186/1472-698X-6-9

**Published:** 2006-07-20

**Authors:** Sekela Mwakyusa, Annah Wamae, Aggrey Wasunna, Fred Were, Fabian Esamai, Bernhards Ogutu, Assumpta Muriithi, Norbert Peshu, Mike English

**Affiliations:** 1 KEMRI Centre for Geographic Medicine Research – Coast, P.O. Box 43640, Nairobi, Kenya; 2 Division of Child Health, Department of Preventive and Promotive Health, Ministry of Health, Nairobi, Kenya; 3 Department of Paediatrics and Child Health, University of Nairobi, Kenyatta National Hospital, Nairobi; 4 Department of Child Health and Paediatrics, College of Health Sciences, Moi University and Moi Teaching and Referral Hospital, Eldoret, Kenya; 5 KEMRI Centre for Clinical Research/USAMRU-K/KEMRI – Kisumu, P.O. Box 54, Kisumu, Kenya; 6 WHO Kenya Country Office, Nairobi, Kenya; 7 KEMRI Centre for Geographic Medicine Research – Coast, P.O. Box 230, Kilifi, Kenya; 8 Department of Paediatrics, University of Oxford and John Radcliffe Hospital, Headington, Oxford, OX3 9DU, UK

## Abstract

**Background:**

The structured admission form is an apparently simple measure to improve data quality. Poor motivation, lack of supervision, lack of resources and other factors are conceivably major barriers to their successful use in a Kenyan public hospital setting. Here we have examined the feasibility and acceptability of a structured paediatric admission record (PAR) for district hospitals as a means of improving documentation of illness.

**Methods:**

The PAR was primarily based on symptoms and signs included in the Integrated Management of Childhood Illness (IMCI) diagnostic algorithms. It was introduced with a three-hour training session, repeated subsequently for those absent, aiming for complete coverage of admitting clinical staff. Data from consecutive records before (n = 163) and from a 60% random sample of dates after intervention (n = 705) were then collected to evaluate record quality. The post-intervention period was further divided into four 2-month blocks by open, feedback meetings for hospital staff on the uptake and completeness of the PAR.

**Results:**

The frequency of use of the PAR increased from 50% in the first 2 months to 84% in the final 2 months, although there was significant variation in use among clinicians. The quality of documentation also improved considerably over time. For example documentation of skin turgor in cases of diarrhoea improved from 2% pre-intervention to 83% in the final 2 months of observation. Even in the area of preventive care documentation of immunization status improved from 1% of children before intervention to 21% in the final 2 months.

**Conclusion:**

The PAR was well accepted by most clinicians and greatly improved documentation of features recommended by IMCI for identifying and classifying severity of common diseases. The PAR could provide a useful platform for implementing standard referral care treatment guidelines.

## Background

A survey conducted to assess the performance of district hospitals in Kenya in the year 2002 demonstrated significant shortcomings in the care of admitted children [1]. One of the problems identified in the survey was that data required for even crude health systems performance indicators were of poor quality and potentially misleading. Such data are likely to prevent effective health care planning for the hospital, region and nation. Several studies have shown that introduction of structured admission forms improve documentation and performance of the health provider [2-4] and that information is both more complete and more concise [5].

Although a structured admission form is an apparently simple measure to improve data quality, poor motivation, lack of supervision, lack of resources and other factors are conceivably major barriers to their successful use in a Kenyan public hospital setting. In Malawi integrated care pathways, in several formats, were successfully introduced by senior clinicians in a teaching hospital setting and these improved record keeping [6]. We wished to examine whether a standardised form would improve the quality of documentation of illness in a typical district hospital in which none of the investigators had any clinical or administrative role. The form aimed to cover common childhood illnesses and is based on the referral care component of the Integrated Management of Childhood Illness (IMCI) strategy. Many of the symptoms and signs included in IMCI are not new to Kenya as they have been part of several long-standing, specific, national and international disease management programmes and guidelines. Data from the pre-intervention period would therefore indicate the degree to which specific disease programmes have influenced documentation of severe illness.

## Methods

### The Paediatric Admission Record (PAR)

The IMCI strategy focuses on the most common childhood conditions that together result in over 80% of paediatric admissions and mortality in most African hospitals: malaria, anaemia, pneumonia, diarrhoea/dehydration, malnutrition, HIV/AIDS, meningitis and the sick young infant. These conditions are defined as IMCI diagnoses and all remaining conditions as non-IMCI diagnoses for the purposes of this report. The design of the PAR reflected both key IMCI symptoms and signs and approaches to severity classification and local information needs identified after meetings with hospital staff. The PAR was therefore administratively accepted as the first stage in the official hospital record and was not a 'study form'. For ease of completion the presence of many symptoms and signs could be indicated by circling options provided, although space was available for more traditional free text. Treatment and nursing observations were recorded on the usual hospital forms.

### Implementation

The Naivasha District Hospital was selected in collaboration with the Division of Child Health in the Ministry of Health for this pilot work as it was reasonably accessible to the study team (based in Nairobi) and provided an anticipated paediatric workload of approximately 1800 admissions per annum. Permission from the hospital management team was sought before embarking on the study which was explained to staff at a subsequent open meeting. We conducted a three-hour training workshop for clinicians and nurses to introduce the PAR. Lectures, discussions and videos were used to explain and demonstrate the symptoms and signs. Attendance was not compulsory and no incentives were offered.

Thirty-two members of staff including 2 medical officers, 6 clinical officers (health workers with 3 years medical training), 18 nurses and 6 others (medical records, X-ray, laboratory and pharmacy staff) were trained during the initial workshop. This initial training included 6/16 of the clinicians who might be responsible for admitting children. Amongst these six were most of those assigned to routine, daytime paediatric services at the time the PAR was introduced. We conducted five more training sessions on the PAR over the study period in order to include all clinical staff involved in the care of children. The total number of staff trained in use of the PAR at the end of the study was 67 including 15 of the 16 clinical officers in the hospital. All training was conducted within the hospital.

At the hospitals request, we also provided 9 hours of training on paediatric inpatient management based around WHO guidelines [7]. At the start of the study only one clinician had received IMCI training, no further staff received IMCI training during the intervention period. Clinical officers responsible for admitting children were each assigned a unique identifying code known only to the research staff at the start of the study.

The study period was divided into pre and post intervention by the PAR introduction. The first PAR training was conducted on 17^th ^May 2004 and the PAR was introduced on 24^th ^May 2004 to replace the existing paediatric medical records. Thus the period before the 17^th ^May 2004 was regarded as the pre-intervention period and from this period we retrospectively selected 168 consecutive paediatric case records over a two months period (from 3^rd ^March 2004 to 3^rd ^May 2004). These records were scrutinized for documentation of symptoms and signs specified in the PAR and data abstracted. We then randomly, prospectively selected 60% of all calendar dates during an 8-month study period aiming for approximately 150 admission events per 2 months and abstracted the same data from the records of children admitted on those dates. This 8 months period is referred to as the post-intervention period. During the post-intervention period feedback on the use of the PAR was provided at an open meeting held every two months where staff had a chance to ask questions and give suggestions. These feedback meetings therefore divided the post-intervention period into four blocks of two months.

In the post-intervention period only data present on the PAR was abstracted. Before intervention the respiratory rate and a history of fever were rarely documented in the clinical record. However, on a number of occasions a measured temperature and a record of respiratory rate were documented by nurses at admission perhaps obviating the clinician's need to include these in the clinical record. We therefore did not assess the completeness of documentation in the pre-intervention period for these specific signs nor for the assessment of conscious level using the AVPU scale (**A**lert, responds to **V**oice, responds to **P**ain, **U**nresponsive) that was not in use before intervention.

Data were entered on site by the investigators using an entry programme with in-built range and consistency checks designed to minimize data entry errors. Personal identifiers were not recorded in this database.

### Analysis

Data were cleaned and analysed using STATA v8.0 (Stata Corporation, USA). The documentation of IMCI emphasized symptoms and signs over time is described both in all records and for specific, common diagnoses. The χ^2 ^test and Odds ratio are used to examine associations between use of the PAR and diagnostic group (IMCI or non-IMCI). The χ^2 ^test for trend and odds ratios are used to examine for an association in the use of the PAR with calendar time. To explore whether the record of the presence or absence of clinical signs on the PAR had clinical significance rather than being a hurried and inaccurate or random record of the clinical consultation we examined whether signs supposed to indicate severe disease were associated with increased mortality. To do this we used computer algorithms based on IMCI symptoms and signs for two common diseases, malaria and pneumonia, to define severe disease based solely on the recorded clinical data. We examined the association of both these derived severity classifications and the clinician's overall diagnosis of the severity of disease with death as the gold standard indicator of severe disease. For the purposes of these analyses pneumonia classifications were dichotomised as very severe or not and malaria as severe or not. This approach provides some indication of the internal validity of the data recorded on the PAR.

### Ethical approval

Ethical approval was obtained from the KEMRI National Ethics Committee and the Kenyan Ministry of Health.

## Results

A total of 868 records were available for analysis, 163 pre and 705 post intervention.

### General characteristics

Before intervention, the number of newborns less than 7 days old (not covered by the current IMCI strategy) was very small (Table 1). During this time most sick babies born in hospital were considered part of the mother's admission and had no separate inpatient number or medical record. Throughout the period of data collection consistently more than 80% of the children admitted had an IMCI diagnosis. Overall mortality was 13% (Table 1).

**Table 1:** Characteristics of children included in the evaluation of medical records.

	Pre-intervention	Post-intervention periods				Total
		1	2	3	4	

Median age (months {IQR})	12 (5–31)	10 (4–21)	2112 (3–28)	11 (4–24)	9 (1–26)	
Newborns (<7d)	5 (3)*	27 (14)	27 (15)	16 (9)	31 (21)	106 (12)
Sex (M)	100 (61)	112 (57)	115 (65)	98 (54)	74 (51)	499 (57)
Malaria	95 (59)	99 (50)	79 (45)	85 (47)	51 (35)	409 (47)
Pneumonia	65 (40)	73 (37)	61 (35)	54(30)	33 (22)	286 (33)
Dehydration	41 (25)	36 (18)	42 (24)	52 (29)	32 (22)	203 (23)
Non IMCI	8 (5)	17 (9)	29 (15)	37 (20)	31 (21)	122 (14)
Mortality	21 (13)	39 (20)	12 (7)	21 (12)	24 (16)	117 (13)
Total	163	200	176	182	147	868

* Figures in parentheses are percentage of the total

Children with more than one diagnosis are represented in more than one group.

### Use of the PAR

**Figure 1:**
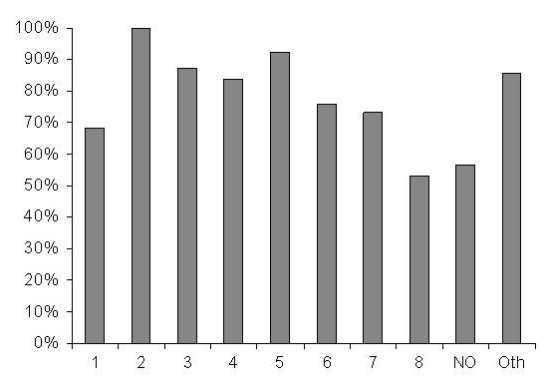
Use of the PAR as the admission record by individual clinicians (Numbered bars 1 to 8 represent data on individual clinicians, NO, data pooled from nursing officers who admit on the nursery and Oth, others, representing a group of clinicians who individually admitted < 20 children each in the post-intervention phase).

Sixty nine percent of the children admitted during the post-intervention study period had a PAR used. The use of the PAR increased over time from 50% (post-intervention period 1) to 84% (post-intervention period 4) at the end of the study [score test for trend, p < 0.001, odds ratio per unit increase in time period, 1.37, 95% CI 1.14–1.63), p < 0.001 (table 2)]. There was considerable variation between clinicians in the use of the PAR ranging from 53% to 100% (Figure 1) and this was unsurprisingly associated with the date of their training (data not shown). Use of the PAR was also found to vary depending on the diagnosis. Forty six percent of children with a non-IMCI diagnosis had a PAR used whereas 73% of children with an IMCI diagnosis had the PAR used [OR 0.32, 95% CI 0.21–0.41, p < 0.001 (Table 2)].

**Table 2:** Use of the paediatric admission record and relationship with diagnosis and time.

	PAR used	Not used	Total
a) By diagnosis			
IMCI	432 (73)	159 (27)	591
Non IMCI	53 (46)	61 (54)	114
			
b) By time			
PI-1	99 (49.5)	101 (50.5)	200
PI-2	127 (72)	49 (28)	176
PI-3	136 (75)	46 (25)	182
PI-4	123 (84)	24 (16)	147
Total	485	220	705

* Figures in parentheses are percentage of the total

### Quality of documentation

There was a marked improvement in documentation of important general clinical features like pallor and level of consciousness for the admitted children. There was less impressive improvement in documentation of others, notably weight and immunization status (from 55 to 58% and 1–21% at the end of the study respectively, table 3). Before the pilot study began signs known to be important in classification of severity of common, specific illnesses [7] were rarely recorded (if at all). During the study we saw a marked improvement in documentation of signs for classifying the severity of malaria, pneumonia and dehydration (table 3). For example the proportion of children admitted with a diagnosis of pneumonia who had a record of the key sign of indrawing increased from 33% in the pre-intervention period to 91% in the final post-intervention period. The fact that a diagnosis specific analysis (that is based on all records, not just those when a PAR was used) shows greater improvement in documentation than the overall analysis suggests that documentation improves if the sign is perceived as directly relevant to the diagnosis.

**Table 3:** Proportion of all records and proportion of records from children admitted with major, common diseases in which clinical features of particular relevance were recorded in the pre-intervention and the four post-intervention periods. The Kenyan Acute Respiratory Infection (ARI) and Control of Diarrhoeal Diseases (CDD) Programmes (now merged in the IMCI programme) have been in place promoting use of WHO case management for more than 10 years. New National Malaria Control Programme guidelines were disseminated in 1998

**All records**	**Kenyan programme (Duration)**	**Pre-intervention (%)**	**Post intervention periods (%)**			
			**One**	**Two**	**Three**	**Four**
Weight		47	22	24	32	41
Record of DTP-HepB-HiB vaccination		1	45	33	32	31
History of Fever	Malaria (6 yrs)		77	82	79	81
History of Convulsions	Malaria, (6 yrs)	20	51	72	70	75
History of Cough	ARI/IMCI (>10 yrs)	53	70	77	73	66
History of Difficulty Breathing	ARI/IMCI (>10 yrs)	37	62	75	70	69
History of Diarrhoea	CDD/IMCI (>10 yrs)	43	58	74	73	72
History of Diarrhoea > two weeks	CDD/IMCI (>10 yrs)	22	48	69	67	59
History of vomiting everything	-	6	47	67	68	71
Conscious level – AVPU classification	-		42	78	65	67
Ability to Drink	Malaria (6 yrs)	3	41	67	66	75
Stiff Neck	-	45	42	69	65	72
Presence of Visible Severe Wasting	-	3	41	68	66	64
Bilateral Oedema of Kwashiorkor	-	31	55	74	71	65
Respiratory Rate	ARI/IMCI (>10 yrs)		64	56	53	59
Lower chest wall indrawing	ARI/IMCI (>10 yrs)	43	49	67	68	67
Cyanosis	ARI/IMCI (>10 yrs)	36	56	75	73	73
Grunting	-	13	42	69	65	68
Acidotic/Deep breathing	Malaria (6 yrs)	3	41	65	64	57
Wheeze		16	36	67	63	57
Sunken Eyes	CDD/IMCI (>10 yrs)	5	43	67	66	58
Skin pinch (skin turgor)	CDD/IMCI (>10 yrs)	3	41	65	65	53
***Records of malaria cases***						
Conscious level – AVPU classification		-	42	79	74	80
Acidotic/Deep breathing	Malaria (6 yrs)	2	44	77	80	78
***Records of Dehydration/diarrhoea cases***						
Sunken Eyes	CDD/IMCI (>10 yrs)	2	60	83	90	83
Skin pinch (skin turgor)	CDD/IMCI (>10 yrs)	7	60	86	92	83
***Records of ARI/Pneumonia cases***						
Lower chest wall indrawing	ARI/IMCI (>10 yrs)	33	69	85	87	91
Ability to Drink	ARI/IMCI (>10 yrs)	4	53	85	85	100

To examine whether the improvement in the quality of documentation was simply a result of the presence of the intervention team (a Hawthorne effect) we analysed the quality of documentation in relation to PAR use in the post-intervention period only. We found that when the PAR was used documentation for signs relevant to specific diagnoses was almost always more than 90% for key signs compared to <50% when clinicians still used traditional free text notes (table 4). The analyses exploring the internal validity of the data recorded on the PAR (whether the data had clinical meaning) showed similar associations with death for the clinician's classification and the computer derived classification that was based on the recorded signs (table 5). This suggests that clinicians were not just mechanically 'ticking the boxes' when completing the PAR to oblige the study team but that the recorded data did have clinical meaning.

**Table 4:** Recording of key, diagnosis-specific, clinical signs in the post-intervention period and association with use of the paediatric admission record.

**Indicator signs.**	**PAR not used**	**PAR used**
***Malaria***	**n = 95**	**n = 219**
AVPU	18 (19%)	188 (86%)
Acidotic breathing	1 (1%)	211 (96%)
***Pneumonia (aged 2–59 m)***	**n = 46**	**n = 175**
Indrawing	13 (28%)	165 (94%)
Ability to drink	0 (0%)	168 (96%)
***Diarrhoea/Dehydration (aged 2–59 m)***	**n = 23**	**n = 139**
Sunken eyes	1 (4%)	129 (93%)
Skin pinch	1 (4%)	131 (94%)

**Table 5:** Association of clinician's classification of severity and the severity classification assigned by a computer algorithm based on data from recorded clinical signs (called the IMCI classification) in cases of pneumonia and malaria from the post-intervention period.

	**Alive**	**Died**	**OR**	**95% CI**
***Malaria – IMCI classification***				
Non-severe	257	18	4.0	1.4 – 10.7
Severe	25	7		
***Malaria – Clinician's classification***				
Non-severe	137	8	2.1	0.9 – 5.0
Severe	148	18		
***Pneumonia – IMCI classification***				
Not very severe	62	6	2.8	1.0 – 7.4
Very severe	75	20		
***Pneumonia – Clinician's classification***				
Not very severe	160	24	1.7	0.6 – 4.9
Very severe	20	5		

## Discussion

One of the objectives of the study was to see whether clinicians would accept the PAR since it was introduced by an external group with no specific incentives and without close supervision. We found that the PAR was in general well accepted and at the end of the pilot study 84% of the children were being admitted using the PAR reflecting a gradual improvement over time plausibly related to both the ongoing training and the 2 monthly, open feedback sessions (Table 2).

There was, however, reasonable variation (53% to 100%) in use of the PAR between clinicians (Figure 1). Factors associated with lower use of the PAR included later training, admission with a non-IMCI diagnosis (e.g. burns or a surgical condition) and clinical cadre. The association with later training may be confounded by a more general problem of poor motivation. Those who delay attending training may be least likely to act on any new initiative. More active supervision might be expected to address this problem. Thus, in a study by Duggan *et al*, observation of primary care providers was found to correlate positively with improved quality of records [2]. Nurses (NO) were in general responsible for admitting newborn babies to the nursery and had a lower rate of use of the PAR (figure 1). Informal comments made suggested that some nurses felt uncomfortable committing themselves to writing the 'medical record', seeing the use of the PAR as the clinician's duty even if the clinician was not expected to see the child until the next day.

There was a marked improvement in documentation over time for most specific disease related signs such as conscious level, chest indrawing, and skin turgor (table 3). As documentation did not improve for these features for children admitted in the post-intervention period when a PAR was not used the improvement can be attributed to the PAR and is in keeping with several studies showing that structured admission forms result in marked improvements in data recording [5,8,9]. The fact that the recorded results of clinical symptoms and signs assessed were predictive of death argues that the PARs were completed meaningfully and that the improved data was of value. However, data on admission weight, immunisation status and respiratory rate were each missing from 40% of records or more even though the data were clearly required in the PAR.

Why does performance vary markedly for individual symptoms and signs? Our study was not designed to address this question. However, we can speculate that several factors may contribute. The majority of symptoms and signs can be evaluated rapidly and the PAR permitted rapid documentation (circling an option). A minimum of effort was therefore required and in fact, informally, many clinicians commented that the PAR made admitting patients quicker, again consistent with work showing that a well designed standardized admission form could reduce the workload of the admission team and length of admission clerking[10]. Counting the respiratory rate or sending the patient out to be weighed, however, require somewhat more effort which alone might result in a failure of documentation. In addition, perhaps these data and that of immunisation status might be regarded as more of a nursing process than a medical process making the clinician happy to ignore them. Failure to record these data may also reflect a perception that they are not important (with which we disagree), or at least not important enough to warrant the additional effort required. Whatever the reason it is possible that a more active process of supervision might address these failings.

Although our results are encouraging there are clearly potential issues of sustainability. Introducing a new inpatient form is associated with some costs. However, the fact that the form is a single sheet of paper used for inpatients alone meant that producing the each form cost only 3 – 4 Kenyan shillings or approximately US$ 50 per 1,000 admissions, even if sustained using a standard photocopy. Costs could be considerably lower if forms were commercially printed. Cost is not the only issue in sustainability. We do not know whether high utilisation rates of the PAR were sustained after the withdrawal of feedback and it may be that some supervision or oversight of the process is required on a continuous basis.

A limitation of this study was that it was done in only one district hospital making it hard to generalise about likely performance elsewhere even though the results are very encouraging. Furthermore improving documentation, while of some value in its own right as an information tool, may have no direct effect on the quality of care offered to children, a far more important outcome. However, as an aide to ensuring a comprehensive clinical review and promoting decisions on disease severity based on evidence the PAR may be a useful first step to improve the care of children admitted to hospital with conditions responsible for most deaths. One disadvantage of a standardised approach, however, is the danger that the resultant focus on specific tasks may limit the breadth and depth of any one child's evaluation.

## Conclusion

In conclusion, we have found that the standardized paediatric admission record was well accepted by clinicians and it greatly improved documentation of illness for most admitted children. This form may be a useful tool in the implementation of the Division of Child Health guidelines for care and IMCI at district hospitals. Future work should consider the possibility of incorporating both clinical and nursing records into a unified format that may further enhance the quality of records and promote efficient use of time.

## Abbreviations

CI – Confidence Interval

IMCI – Integrated Managemnt of Childhood Illness

OR – Odds Ratio

PAR – Paediatric admission record

WHO – World Health Organisation

## Competing interests

The author(s) declare that they have no competing interests.

## Authors' contributions

Dr. Sekela Mwakyusa was responsible for undertaking the study, collecting the data, data management, analysis and writing the manuscript.

Dr. Annah Wamae, Prof. Aggrey Wasunna, Dr. Fred Were, Prof. Fabian Esamai, Dr. Bernhards Ogutu, Dr. Assumpta Muriithi, and Dr. Norbert Peshu contributed to the design of the study, oversaw its implementation, reviewed the analysis and contributed to drafting of the paper.

Dr. Mike English designed the study, obtained funding and supervised data collection, data management, analysis and the writing of the manuscript.

## Pre-publication history

The pre-publication history for this paper can be accessed here:


